# Supplementary material for: Chimpanzee groups achieve sustainable resource use in a common-pool resource dilemma
Source: Commun Psychol. 2026 Jan 17;4:22. doi: 10.1038/s44271-025-00390-8 (PMC12876908; doi:10.1038/s44271-025-00390-8)
Supplement: Supplementary file 2 — Supplementary Information: Chimpanzee groups achieve sustainable resource use in a Common-Pool Resource dilemma [file 44271_2025_390_MOESM2_ESM.docx]

Supplementary Information

Chimpanzee groups achieve sustainable resource use in a Common Pool Resource dilemma

Kirsten Sutherland^1^, Daniel Haun*^1^, Alejandro Sánchez-Amaro*^1,2^

^1^Max Planck Institute for Evolutionary Anthropology

^2^University of Stirling

* shared last authorship

Table of Contents

[1. Deviations from preregistration 1](#_Toc187677447)

[1.1 Hypotheses 1](#_Toc187677448)

[1.2 Analysis 3](#_Toc187677449)

[2. Experimental methods 3](#_Toc187677450)

[2.1 Testing group information 3](#_Toc187677451)

[2.2 Pretest experience and comprehension criteria 6](#_Toc187677452)

[2.3 Data recording and coding methods 9](#_Toc187677453)

[2.4 Dropped trials and coding errors 12](#_Toc187677454)

[2.5 Trial cutoffs 12](#_Toc187677455)

[2.6 Reliability coding 13](#_Toc187677456)

[3. Statistical methods 13](#_Toc187677457)

[3.1 Model predictive power comparisons 13](#_Toc187677458)

[3.2 Full model summaries 14](#_Toc187677459)

[3.2.1 Model 1 14](#_Toc187677460)

[3.2.2 Model 2 (dyads) 15](#_Toc187677461)

[3.2.3 Model 3 (quartets) 16](#_Toc187677462)

[3.2.4 Model 4 (quartets) 16](#_Toc187677463)

[3.2.5 Model 5 (dyad) 17](#_Toc187677464)

[3.2.6 Model 6 19](#_Toc187677465)

[3.2.7 Model 7 (dyads) 20](#_Toc187677466)

[3.2.8 Model 8 (quartets) 20](#_Toc187677467)

[3.3 Other statistical methods 21](#_Toc187677468)

[4. Intervention behaviours 21](#_Toc187677469)

[4.1 Stick guarding 21](#_Toc187677470)

[4.2 Physical interventions 22](#_Toc187677471)

[4.3 Vocal interventions 22](#_Toc187677472)

[4.3 Stick grabbing 23](#_Toc187677473)

# Deviations from preregistration

The method, analysis plan, and hypotheses were registered prior to data collection (<https://osf.io/h7245>), on 14th October 2022. Places where the study deviated from the original plan are detailed below.

## Hypotheses

Hypothesis 3 was preregistered as “groupings with heterogenous dominance will be more successful than those with similarly-matched dominance rankings. This effect will be greater in smaller groups”. The text in the preregistration continues, describing the success of low social tolerance dyads found in Koomen & Herrmann (2018). Prior to the completion of data collection and prior to any analysis or data visualisation, the hypothesis was corrected to explicitly state the expected importance of tolerance: “groups with heterogenous dominance and low social tolerance will be more successful than those with similarly-matched dominance rankings. This effect will be greater in dyads.”. A similar change was made to hypothesis 6 in the preregistration (hypothesis 4 in the main text). The original hypothesis stated “payoffs will be more asymmetrical in dyadic groups than in quartets. This effect will be enhanced in groups that also have asymmetrical dominance”. This was changed to: “payoffs will be more unequal in dyadic groups than in quartets, and that this effect will be enhanced in groups that had asymmetrical dominance and low social tolerance”.

Hypotheses 4 and 5 from the preregistration were not included in the main text in an effort streamline the paper and reduce word count. These hypotheses were still tested as planned and results are detailed below. The hypothesis that is registered as “hypothesis 6” in the preregistration is therefore refered to as “hypothesis 4” in the main text.

Hypothesis 4 was registered as “performance of groups will improve over sessions as a learning effect”. No clear effect of session, or its interaction with condition, was identified in models 1, 2 and 3. Therefore, hypothesis 4, that latency would increase over sessions as a learning effect, is not supported.

Hypothesis 5 was preregistered as “The proportion of participants interacting with the apparatus will be lowest in dyadic groups with heterogenous dominance”. To test this, a GLMM (model 9) tested the effect of *Group Size*, Group Dominance Difference, and Group Social Tolerance on *participation*. *Participation* was a proportion that reflected the number of individuals in a testing group that interacted with the apparatus by touching a stick during a given trial. As the possible values that *participation* could take were limited to 0.25, 0.50, 0.75 and 1.00, the response variable was treated as ordinal and used a cumulative logit link function. Normal and weakly informative priors with a mean of 0 and standard deviation of 1 were used for the fixed effects (*Group Size*, *Group Dominance Difference*, and *Group Social Tolerance*). As the *Participation* measure was calculated from stick-holding durations, the model was fitted to the test condition trials where accurate durations were codable (*N*=368).

Model 9 (output details in table S1) found a negative effect of group size on participation proportion, suggesting that participation was significantly lower in quartets, not dyads (-2.21, 95%CI[-2.88, -1.51]). This is because, in dyads, it was much more likely that both participants would interact with the apparatus rather than just one. This is also related to the average shorter latencies until collapse in the dyads. In quartets, the modal participation proportion was 0.75, compared to 1.0 in dyads. No effects of *Group Dominance Difference*, *Group Social Tolerance ,* or their interactions were identified. A negative interaction with an effect size -1.00 and a 95% CI almost excluding zero ([-2.05, 0.20]) was identified between tolerance and group size. Posterior probability of a negative interaction was 94.75%. This suggests that participation is lower in quartets, and even more so when tolerance is high.

Model 9 formula:

*Participation Proportion* ~ *Group Dominance Difference* * *Group Social Tolerance* * *Group Size* + (1| *Group ID*) + (1 | *Player name*)

| **Parameter** | **Estimate** | **Est. Error** | **95% CI (l, u)** | **Probability of direction (%)** | **Rhat** | **ESS** |
| --- | --- | --- | --- | --- | --- | --- |
| Intercept [1] | -2.28 | 0.23 | -2.74, -1.84 | 100 | 1.00 | 2446 |
| Intercept [2] | -0.97 | 0.20 | -1.37, -0.58 | 99.98 | 1.00 | 3570 |
| *Group Dominance Difference* | 0.08 | 0.27 | -0.45, 0.62 | 63.10 | 1.00 | 2597 |
| *Group Social Tolerance* | -0.31 | 0.31 | -0.90, 0.34 | 84.65 | 1.00 | 2377 |
| *Group Size* | -2.21 | 0.34 | -2.88, -1.51 | 100 | 1.00 | 2684 |
| *Group Dominance Difference: Group Social Tolerance* | 0.58 | 0.42 | -0.27, 1.40 | 91.83 | 1.00 | 2872 |
| *Group Dominance Difference: Group Size* | -0.33 | 0.48 | -1.29, 0.63 | 76.35 | 1.00 | 3035 |
| *Group Social Tolerance*: *Group Size* | -1.00 | 0.57 | -2.05, 0.20 | 94.75 | 1.00 | 2601 |
| *Group Dominance Difference: Group Social Tolerance*: *Group Size* | 0.55 | 0.72 | -0.86, 1.95 | 77.65 | 1.00 | 3080 |

Table S1: Model 9 output summary table

## Analysis

Model 1 in the main text is labled “model 2” in the preregistration. Models 2 and 3 in the main text are both labled “model 1” in the preregistration. Other than these name changes, the models were fitted as described in the preregistration.

# Experimental methods

## Testing group information

Table S2 shows testing group information. In the majority of Fraukje’s tests, she was joined by her 2 year old daughter Carola. Carola was not counted as a participant as she was too young to participate in the comprehension test. Carola never removed a stick from the pool. She was small enough to fit her arm through the metal mesh and collect yoghurt directly with her fingers. Although in theory she could have held the lid open for the larger apes, there were no observations of Carola being used as a social tool (such as in Schweinfurth et al., 2018; Völter et al., 2015).

| Group ID | Particpants (number indicates relative rank, 1 is most dominant) | Dominance difference | Group social tolerance |
| --- | --- | --- | --- |
| Dyads | | | |
| D1 | 1. Frodo  2. Riet | 1 | 0.03690037 |
| D2 | 1. Swela  2. Azibo | 4 | 0.1909385 |
| D3 | 1. Bambari  2. Azibo | 6 | 0.05050505 |
| D4 | 1. Frodo  2. Maja | 7 | 0.02439024 |
| D5 | 1. Swela  2. Maja | 2 | 0.07142857 |
| D6 | 1. Changa  2. Fraukje | 4 | 0.03103448 |
| D8 | 1. Sandra  2. Fraukje | 2 | 0.01666667 |
| D9 | 1. Changa  2. Azibo | 5 | 0.05617978 |
| D10 | 1. Maja  2. Fraukje | 1 | 0.0896861 |
| D11 | 1. Riet  2. Changa | 3 | 0.2011173 |
| D12 | 1. Taï  2. Sandra | 4 | 0.01465201 |
| D13 | 1. Frodo  2. Swela | 5 | 0.03433476 |
| BD1 | 1. Alex  2. Hope | 1 | 0.1454545 |
| BD2 | 1. Frederike  2. Zira | 1 | 0.01932367 |
| BD3 | 1. Hope  2. Zira | 2 | 0.3311258 |
| BD4 | 1. Daza  2. Frederike | 2 | 0.09767442 |
| BD6 | 1. Hope  2. Daza | 1 | 0.03921569 |
| Quartets | | | |
| Q1 | 1. Frodo  2. Riet  3. Swela  4. Azibo | 4 | 0.0796441417 |
| Q3 | 1. Sandra  2. Swela  3. Maja  4. Fraukje | 1 | 0.0338773422 |
| Q4 | 1. Riet  2. Taï  3. Sandra  4. Fraukje | 5 | 0.036924983 |
| Q5 | 1. Changa  2. Maja  3. Fraukje  4. Azibo | 3 | 0.0406629167 |
| Q6 | 1. Riet  2. Taï  3. Changa  4. Sandra | 3 | 0.082832276 |
| BQ1 | 1. Alex  2. Hope  3. Zira  4. Frederike | 3 | 0.1146764 |
| BQ2 | 1. Alex  2. Hope  3. Daza  4. Frederike | 2 | 0.100423075 |

Table S2: Testing group information

## Pretest experience and comprehension criteria

| **Condition** | **Photo** | **Stick positions** |
| --- | --- | --- |
| Pre-experience | 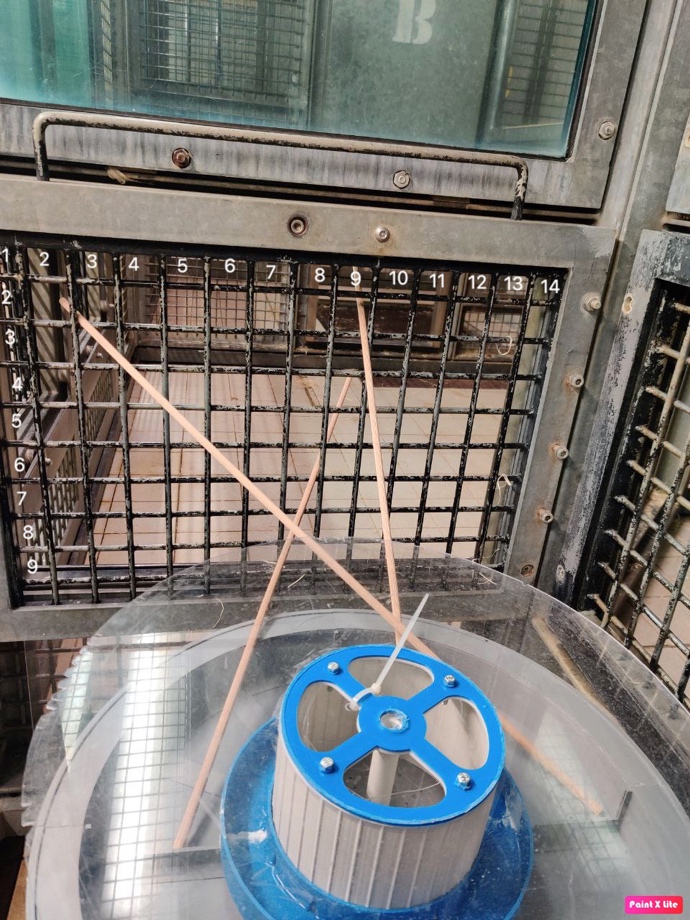 | Left grid: 3,2 and 9,2 and 8,6  Middle grid: none  Right grid: none |
| Dyad | 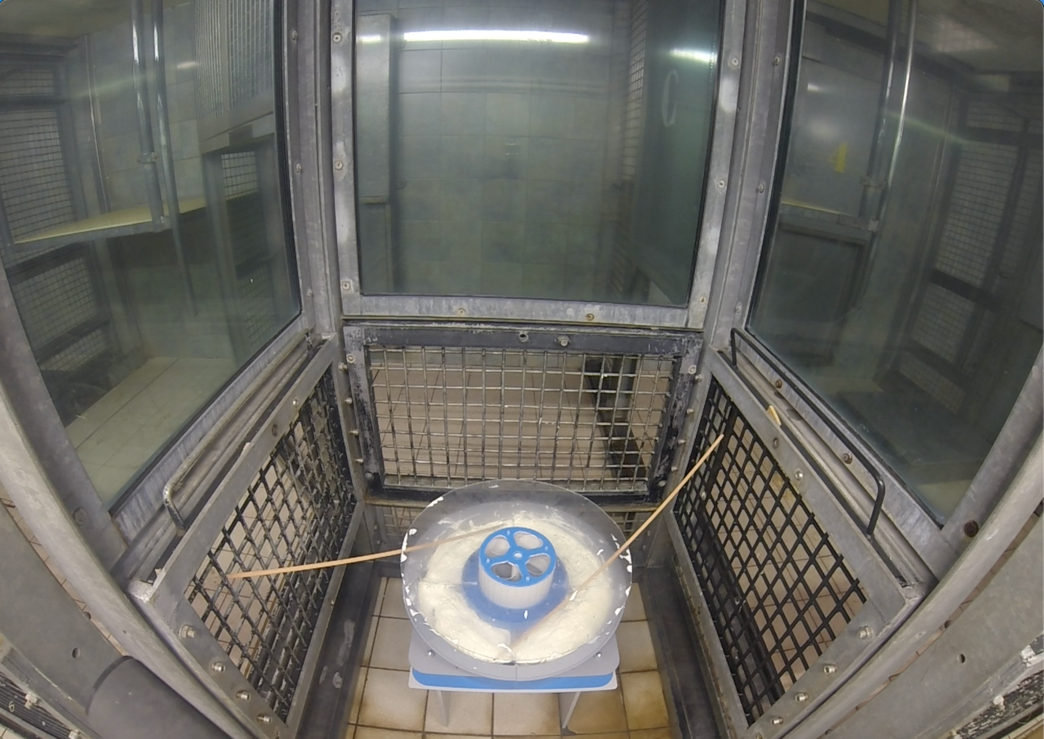 | Left grid: 3,2  Middle grid: none  Right grid: 3,2 |
| Quartet | 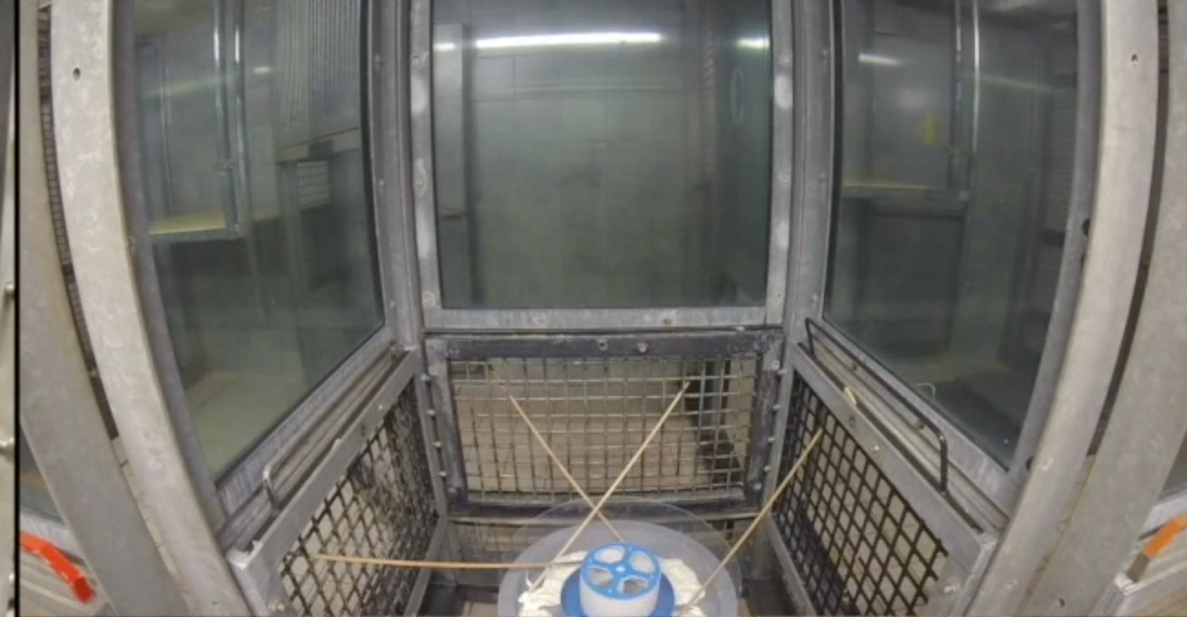 | Left grid: 3,2  Middle grid: 4,3 and 13,3  Right grid: 3,2 |

Table S3: Stick starting positions

Pre-experience was carried out with only one individual at a time, to remove the social dilemma. The target participant was called in. The yoghurt-pool apparatus was already set up in the middle of the testing booth. The participant was held in a section of the testing room where they could see the apparatus but not interact with it. The participant was able to observe while the experimenter inserted three sticks at various heights (see table S2), with one stick holding the lid in its highest position. If the participant removed all of the sticks without replacing them, the lid would slip to the bottom of the apparatus, where it could not be pushed back up. This represented system collapse resulting from over-exploitation. A pre-experience session ended, either when the system was collapsed (the lid fell to the bottom, making the yoghurt inaccessible) or after 10 minutes had elapsed. If a participant was successful in two consecutive pre-experience sessions, indicating comprehension of the collapse mechanism, they did not receive any more pre-experience sessions. Success was defined as follows: an individual maintains the system for over 2 minutes (starting when they first touch a stick), provided that they have previously (in the current session OR a previous session) removed the top stick and observed the lid to descend, and fed from the stick at least once. The arrangement of sticks for the conditions are outlined in table S3. The experimenter started the two-angle camera video recording and left the room, observing through the partly-open door and averting their gaze if the participant looked at them. The keeper opened the door inside the enclosure, allowing the participant to enter and access the sticks. The participant was then able to feed from the yoghurt-pool by dipping with the sticks.

Pre-experience was carried out in three phases. Phase 1 had up to 5 “Normal” sessions, with the procedure as described above. If a participant did not remove the top supporting stick in the first three pre-experience sessions, thereby never witnessing the mechanism by which the lid closes, they were given a “forced movement” session, where the supporting stick was accessible only to the experimenter. The experimenter would ensure they have the ape’s attention, before removing the stick and showing them how the lid moves.

Phase 2 was conducted if a participant failed to maintain the resource for two minutes in three consecutive sessions in the “Normal” condition. The first session of Phase 2 was a “forced reinsert” session, where the top stick was inaccessible. In this condition it was impossible to collapse the apparatus. If the participant did not spontaneously reinsert their sticks, they would be coaxed by the experimenter. The session was ended 10 minutes after the first stick was touched. In the following session the participant was given the “packet” condition. The packet would start with one Normal condition. If the Normal condition was failed, the participant would immediately be given a short 2-minute Forced Reinsert condition. After the two minutes they would be given a second Normal condition. If the first Normal condition was successful, the rest of the packet would not be given. The following session would be Normal.

If this Normal session was a failure, then Phase 3 would be initiated, where the participant was given sessions that follwed the Normal procuedure, but with five sticks, rather than three. If two five-stick conditions were successful, then the number of sticks would be reduced back to three. The participant would then be offered a maximum of four Normal conditions. If two consecutive sessions were successful, they would pass the comprehension criteria. Otherwise, they were dropped as participants.

As shown in table S4, 10 out of 15 of participants achieved comprehension criteria in Phase 1. Three participants that finished pre-experience over a month before the other apes were given one refresher session in the Normal condition, which they all passed (maintained for >2 minutes without collapsing).

| Name | Sex | | Age at start of testing | | Number of P-E sessions until pass | Phase reached before pass |
| --- | --- | --- | --- | --- | --- | --- |
|  | |  | | A-Group | | |
| Azibo | Male | | 7 | | 11 | Phase 3 |
| Bambari | Female | | 22 | | 13 | Phase 3 |
| Changa | Female | | 11 | | 3 | Phase 1 |
| Fraukje | Female | | 46 | | 3 | Phase 1 |
| Frodo | Male | | 29 | | 3 | Phase 1 |
| Maja | Female | | 36 | | 3 | Phase 1 |
| Riet | Female | | 45 | | 9 | Phase 3 |
| Sandra | Female | | 29 | | 3 | Phase 1 |
| Swela | Female | | 27 | | 12 | Phase 3 |
| Tai | Female | | 20 | | 7 | Phase 2 |
|  | |  | | B-Group | | |
| Alex | Male | | 21 | | 3 | Phase 1 |
| Daza | Female | | 36 | | 3 | Phase 1 |
| Fredericke | Female | | 48 | | 3 | Phase 1 |
| Hope | Female | | 32 | | 3 | Phase 1 |
| Zira | Female | | 25 | | 4 | Phase 1 |

Table S4: Participant information and sessions until comprehension criteria reached

## Data recording and coding methods

Videos of experimental trials were collected from four angles, with three cameras pointed at the apparatus in the testing room and one capturing the adjoining room that the apes could move freely to. All data were coded from video in Mangold INTERACT. No live-coding took place. The INTERACT ethogram of codable behaviours is detailed in Table S5:

| Code | Description | Start coding when | Stop coding when | Duration? | Class |
| --- | --- | --- | --- | --- | --- |
| Holding s1 | Focal holds stick 1 in their hand, foot, or mouth | The frame when the focal grasps the stick (can be identified looking at the moment the stick moves, usually looks like a “twitch”).  If an ape grasps a stick from the mesh, but does not pull it out of the mesh, do not code it as holding. Only code if the stick is removed.  Sometimes apes will pick up a stick off the floor, rather than removing it from the mesh. Stick identification should be based the stick’s original position (e.g. stick 1(s1) is the one that starts in the farthest left position. The stick is still s1 if an ape carries it around to the right side and drops it on the floor. | The stick leaves the focal’s hand OR the moment the lid closes. | Y | Stick behaviour |
| Holding s2 | Focal holds stick 2 in their hand, foot, or mouth |  | The stick leaves the focal’s hand OR the moment the lid closes. | Y | Stick behaviour |
| Holding s3 | Focal holds stick 3 in their hand, foot, or mouth |  | The stick leaves the focal’s hand OR the moment the lid closes. | Y | Stick behaviour |
| Holding s4 | Focal holds stick 4 in their hand, foot, or mouth |  | The stick leaves the focal’s hand OR the moment the lid closes. | y | Stick behaviour |
| Grab s1 | Focal appropriates stick 1 from another player or attempts to grab it from them | The moment the focal grasps a stick that someone else is holding (code even if the focal does not successfully appropriate the stick).  If the stick is successfully appropriated, the focal is now “holding” the stick, start the Holding s1/2/3/4 code from the moment the “grab” occurs. Likewise, end the holding code for the ape who lost the stick. |  | N | Stick behaviour |
| Grab s2 | Focal appropriates stick 2 from another player or attempts to grab it from them |  |  |  |  |
| Grab s3 | Focal appropriates stick 3 from another player or attempts to grab it from them |  |  |  |  |
| Grab s4 | Focal appropriates stick 4 from another player or attempts to grab it from them |  |  |  |  |
| Lid close | The moment the full circumference of the lid touches the rim of the bowl | The frame where the lid stops moving (or as close as possible). |  | N | Lid |
| Guarding s1 | Focal places their hand on stick 1 still inside mesh (NOT moving it around to try and gather yoghurt. Simply holding it in place) |  |  | y | Intervention behaviour |
| Guarding s2 | Focal places their hand on stick 2 still inside mesh |  |  | y | Intervention behaviour |
| Guarding s3 | Focal places their hand on stick 3 still inside mesh |  |  | y | Intervention behaviour |
| Guarding s4 | Focal places their hand on stick 4 still inside mesh |  |  | y | Intervention behaviour |
| Intervention vocalisation | Focal vocalises in response to another player taking a stick |  |  | n | Intervention behaviour |
| Physical intervention | Focal touches or obstructs another player taking a stick |  |  | n | Intervention behaviour |
| Eat from stick | Focal eats yoghurt from stick (count) | Each stick-mouthful should only be coded once. If an ape dips the stick in yoghurt, and then eats from the stick, press “e” (approximately) when the stick is in their mouth/ their tongue is on the stick. If they remove the stick from their mouth, and then lick it/ put it back in their mouth without dipping it back in the yoghurt, do not press “e” again. Only one mouthful per yoghurt dip should be coded. |  | n | eating |
| Eat from mesh | Focal eats yoghurt from mesh or floor using finger, tongue, etc | Code every time you see the focal lick the mesh or floor, even if you can’t see yoghurt in the spot they are licking from. |  | n | eating |
| start | Doors open to begin test |  |  |  |  |

Table S5: Video coding ethogram

## Dropped trials and coding errors

Four trials were discarded due to experimenter error. A further five trials (outlined in table S6) were associated with error or ambiguity, but were judged to still be usable. Therefore, all videos were coded according to the ethogram and the methods described in the paper, with the following exceptions:

| **Group ID** | **Date of test** | **Trial type and number** | **Details** |
| --- | --- | --- | --- |
| D8 | 24.06.22 | Test 1 | In a long trial where the participants appeared to have lost interest, the trial was mistakenly ended before the cutoff time by the experimenter. Lid latency was coded as the time the trial was ended. |
| D12 | 28.08.22 | Control 6 | Tai brought a stick from outside into the test and attempted to use it to get yoghurt. The stick was too short and she quickly discarded it and took a stick from the pool. |
| D5 | 02.12.22 | Control 8 | One of the internal doors was accidentally kept shut at the start of the test, blocking the apes from accessing one of the sticks. The internal door was opened after a few seconds and, as no sticks were removed before this time, the start of the trial was coded as the time the internal door opened. |
| BQ1 | 17.04.23 | Control 13 | A stick was knocked out but may have still been accessible, before it clearly rolled out of reach. From the camera angle it was difficult to judge the moment it became inaccessible, so it was coded as inaccessible from the moment it was knocked out of the mesh. |
| Q4 | 20.08.22 | Control 1 | A stick was knocked out but may have still been within reach. It was coded as out of reach although it was difficult to judge. |

Table S6: Coding and experimental errors

## Trial cutoffs

In the interest of the apes’ wellbeing, we avoided keeping unmotivated participants inside the testing room. A trial cutoff was therefore imposed if:

1. 10 minutes after the start of the trial, only one player had interacted with the experiment (by touching or licking the sticks or mesh).
2. There was 10 minutes of inactivity (no participant touched or licked the sticks or mesh for 10 minutes).

Eight trials met this criteria. In these trials, the lid collapse and last stick latencies were recorded as 10 minutes, or 10 minutes from the point of inactivity, so they could be included in models 1-3.

## 2.6 Reliability coding

A Pearsons R correlation was used to test the relibility of the two coders’ lid-closing latencies. Inter-rater reliability was excellent with *R*=0.9997. The same test was used to compare the two raters’ codes of stick holding latencies. For stick 1, *r*=0.2925. For stick 2, *r*=0.999. For stick 3, *R*=0.9600. For stick 4, *r*=9999. The lower reliability for stick 1 was caused by the second coder failing to input a cutoff time in one trial. When this trial was removed from analysis, R was 0.9987.

Cohen’s kappa was used to assess inter-rater reliability of yoghurt-eating counts. Very strong agreement between raters was found (*k* =0.865, *z*=19.2, *p*=<0.001).

# Statistical methods

## 3.1 Data availability

The scripts that were used for the analysis, including the initial power analysis, and the raw data can be found in the OSF data repository (<https://osf.io/q8ga6/>).

## 3.2 Model predictive power comparisons

As several high Pareto-*k* values were identified in Model 1 and 2, *k*-fold cross-validation was used instead of Leave One Out – Information Criterion for model comparisons. Every full model was compared to a null model containing only random effects. To avoid over-fitting to group-specific patterns, an argument for a group-based cross-validation was added.

The *k*-fold comparison for model 1 (group size comparison) tended to favour the predictive power of the full model (ELPD_MODEL1_ – ELPD_NULL1_ = -5.1, SE=10.4), while model 3 (quartet condition comparison) clearly favoured the full model (ELPD_MODEL3_ – ELPD_NULL3_ = -16.7, SE=5.6). Model 2 (condition comparison dyad) was not clearly distinguished from the null model (ELPD_NULL2_ – ELPD_MODEL2_ = -60.3, SE=6.8). The *k-*fold comparison for Model 4 (examining relative payoffs, dominance, and tolerance among quartets) showed a preference for the full model (ELPD_MODEL4_ – ELPD_NULL4_ = -4.3, SE=6.1). Model 5 (examining relative payoffs, dominance, and tolerance among dyads) showed a strong preference for the full model ELPD_MODEL5_ – ELPD_NULL5_ = -211.7, SE=18.7), as did model 6 (examining payoff equality in quartets versus dyads) (ELPD_MODEL6_ – ELPD_NULL6_ = -88.2, SE=17.0). Comparisons for models 7 (relationship between payoff equality, dominance and tolerance in dyads) did not distinguish between the full and null models (ELPD_NULL7_ – ELPD_MODEL7_ = -11.1, SE=2.3. Model 8 (relationship between payoff equality, dominance and tolerance in quartets) favoured the full model ELPD_MODEL8_ – ELPD_NULL8_ = -8.1, SE=3.2).

## Full model summaries

The model outputs of the analyses discussed in the main text are summarised in tables in this ection. Tables were made using the report package in R (Makowski et al., 2023). The tables report the median of the posterior distribtion and its 95% Highest Posterior Density Interval and probability of direction (pd). Convergence and stability of the Bayesian sampling has been assesed using R-hat, which should be below 1.01(Vehtari et al., 2021) and Effective Sample Size (ESS), which should be greater than 1000 (Bürkner, 2017). Results discussed in the main text are highlighted.

### Model 1

Addressing hypothesis 1:

Quartets will have more difficulty than dyads in sustaining the CPR, resulting in shorter collpse latencies.

Formula:

*Collapse Latency ~* 1 + *Group Size* * *Session Number* + *Group Size * Group Dominance Difference* + *Group Size* * *Group Social Tolerance* + *Group Dominance Difference* * *Group Social Tolerance* + (1 + *Session Number* | *Group ID*) + (1 | *Player Name*)

Shape ~ 1 + *Group Size*

| **Parameter** | **Estimate** | **Est. Error** | **95% CI** | **Probabiliy of direction (%)** | **Rhat** | **ESS** |
| --- | --- | --- | --- | --- | --- | --- |
| Intercept | 4.67 | 0.14 | 4.39, 4.95 | 100 | 1.00 | 2006 |
| Shape intercept | 0.43 | 0.08 | 0.27, 0.58 | 100 |  | 3683 |
| *Group Size* | 0.86 | 0.27 | 0.31, 1.37 | 99.80 | 1.00 | 1877 |
| *Session number* | -0.03 | 0.08 | -0.18,0.13 | 67.58 | 1.00 | 2361 |
| Group dominance difference | 0.02 | 0.17 | -0.31, 0.34 | 55.95 | 1.00 | 1933 |
| Group social tolerance | -0.03 | 0.20 | -0.43, 0.36 | 55.83 | 1.00 | 2054 |
| Condition:Session | -0.07 | 0.15 | -0.38, 0.21 | 67.92 | 1.00 | 2208 |
| Condition: Group dominance difference | 0.28 | 0.28 | -0.26, 0.83 | 83.62 | 1.00 | 2333 |
| Condition: Group social tolerance | 0.22 | 0.36 | -0.49, 0.91 | 73.35 | 1.00 | 2509 |
| Group dominance difference: Group social toelrance | -0.02 | 0.16 | -0.33, 0.30 | 54.17 | 1.00 | 2474 |
| Shape condition | -0.09 | 0.15 | -0.40, 0.20 | 73.05 | 1.00 | 5428 |

|  |
| --- |
| \|  \| \| --- \| |

Table S7: Model 1 summary table

### Model 2 (dyads)

Addressing hypothesis 2:

Participants will approach and extract from the common pool more quickly in the absence of the social dilemma (i.e. in the control condition).

Addressing hypothesis 3:

Groups with heterogenous dominance and low social tolerance will be more successful than those with similarly-matched dominance rankings. This effect will be greater in dyads.

Formula:

*Last Stick Latency ~* 1 + *Coundition* * *Session Number + Condition* * *Group Dominance Difference* + *Condition* * *Group Social Tolerance* + *Group Dominance Difference* * *Group Social Tolerance* + (1 + *Condition* + *Session Number* | *Group ID*) + (1 | *Player name*)

Shape ~ 1 + *Condition*

| **Parameter** | **Estimate** | **Est. Error** | **95% CI (l, u)** | **Probability of direction (%)** | **Rhat** | **ESS** |
| --- | --- | --- | --- | --- | --- | --- |
| Intercept | 3.88 | 0.20 | 3.48, 4.28 | 100 | 1.00 | 2272 |
| Shape intercept | 0.21 | 0.06 | 0.09, 0.33 | 99.95 | 1.00 | 2839 |
| *Condition* (control, test) | 0.26 | 0.16 | -0.08, 0.57 | 93.80 | 1.00 | 2586 |
| *Session Number* | 0.01 | 0.11 | -0.20, 0.24 | 52.73 | 1.00 | 2656 |
| *Group Dominance Difference* | -0.17 | 0.17 | -0.50, 0.18 | 84.03 | 1.00 | 2416 |
| *Group Social Tolerance* | -0.15 | 0.16 | -0.46, 0.16 | 83.50 | 1.00 | 2642 |
| *Condition* : *Session Number* | 0.12 | 0.09 | -0.06, 0.30 | 90.67 | 1.00 | 2823 |
| *Condition:* *Group Dominance* Difference | -0.06 | 0.13 | -0.32, 0.21 | 69.15 | 1.00 | 3171 |
| *Condition:* *Group Social Tolerance* | -0.11 | 0.13 | -0.37, 0.16 | 79.97 | 1.00 | 2649 |
| *Group Dominance Difference: Group Social Tolerance* | -0.10 | 0.21 | -0.50, 0.32 | 67.92 | 1.00 | 3015 |
| Shape condition ( | -0.26 | 0.13 | -0.51, -0.01 | 97.80 | 1.00 | 2917 |

Table S8: Model 2 summary table

### Model 3 (quartets)

Addressing hypothesis 2:

Participants will approach and extract from the common pool more quickly in the absence of the social dilemma (i.e. in the control condition).

Addressing hypothesis 3:

Groups with heterogenous dominance and low social tolerance will be more successful than those with similarly-matched dominance rankings. This effect will be greater in dyads.

Formula:

*Last Stick Latency ~* 1 + *Coundition* * *Session Number + Condition* * *Group Dominance Difference* + *Condition* * *Group Social Tolerance* + *Group Dominance Difference* * *Group Social Tolerance* + (1 + *Condition* + *Session Number* | *Group ID*) + (1 | *Player name*)

Shape ~ 1 + *Condition*

| **Parameter** | **Estimate** | **Est. Error** | **95% CI (l, u)** | **Probability of direction (%)** | **Rhat** | **ESS** |
| --- | --- | --- | --- | --- | --- | --- |
| Intercept | 4.75 | 0.13 | 4.48, 4.98 | 100 | 1.00 | 1847 |
| Shape intercept | 0.24 | 0.09 | 0.06, 0.42 | 99.35 | 1.00 | 2455 |
| *Condition* (control, test) | 0.70 | 0.17 | 0.37, 1.02 | 99.95 | 1.00 | 2304 |
| *Session Number* | -0.07 | 0.10 | -0.25, 0.14 | 76.75 | 1.00 | 2058 |
| *Group Dominance Difference* | 0.17 | 0.19 | -0.20, 0.54 | 81.67 | 1.00 | 2787 |
| *Group Social Tolerance* | 0.61 | 0.27 | -0.00, 1.08 | 97.47 | 1.00 | 2046 |
| *Condition* : *Session Number* | 0.04 | 0.13 | -0.22, 0.29 | 62.20 | 1.00 | 2747 |
| *Condition:* *Group Dominance* Difference | 0.07 | 0.23 | -0.38, 0.51 | 62.82 | 1.00 | 2449 |
| *Condition:* *Group Social Tolerance* | -0.30 | 0.32 | -0.92, 0.34 | 83.47 | 1.00 | 2843 |
| *Group Dominance Difference: Group Social Tolerance* | -0.49 | 0.36 | -1.16, 0.28 | 91.25 | 1.00 | 2209 |
| Shape condition ( | -0.17 | 0.18 | -0.53, 0.19 | 82.03 | 1.00 | 2928 |

Table S9: Model 3 summary table

### Model 4 (quartets)

Exploration of dominance:tolerance interaction from Model 3.

Formula:

*Collapse Latency ~* 1 + *Proportion* * *Group Dominance Difference* * *Group Social Tolerance* + (1 | *Group ID*) + (1 | *Player name*)

| **Parameter** | **Estimate** | **Est. Error** | **95% CI (l, u)** | **Probability of direction (%)** | **Rhat** | **ESS** |
| --- | --- | --- | --- | --- | --- | --- |
| Intercept | 5.03 | 0.13 | 4.77, 5.28 | 100 | 1.00 | 2972 |
| Shape intercept | 0.32 | 0.13 | 0.06, 0.58 | 99.22 | 1.00 | 2705 |
| *Proportion* | -0.18 | 0.10 | -0.37, 0.03 | 95.73 | 1.00 | 3185 |
| *Group Dominance Difference* | 0.01 | 0.15 | -0.29, 0.33 | 54.10 | 1.00 | 2760 |
| *Group Social Tolerance* | 0.05 | 0.15 | -0.22, 0.36 | 64.55 | 1.00 | 2834 |
| *Proportion* : *Group Dominance Difference* | -0.17 | 0.13 | -0.43, 0.08 | 91.33 | 1.00 | 2894 |
| *Proportion* : *Group Social Tolerance* | -0.06 | 0.12 | -0.28, 0.18 | 69.97 | 1.00 | 3388 |
| *Group Dominance Difference: Group Social Tolerance* | -0.27 | 0.15 | -0.58, 0.03 | 96.38 | 1.00 | 2443 |
| Proportion : Group *Dominance Difference: Group Social Tolerance* | -0.01 | 0.14 | -0.30, 0.26 | 51.75 | 1.00 | 3007 |

Table S10: Model 4 summary table

The highest ranked ape in the group did not necessarily take a stick first (see figure S1).

**
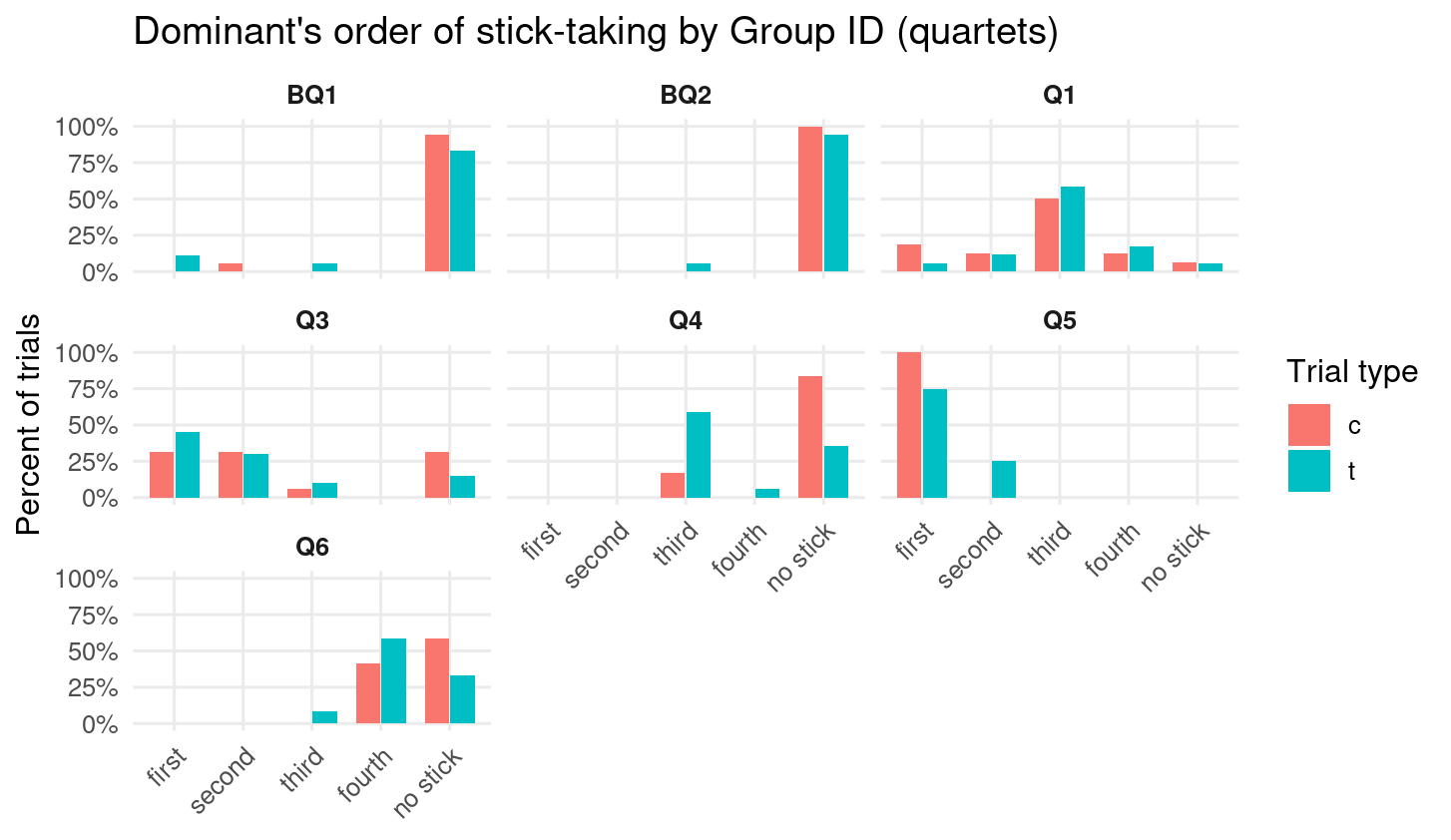
**

Figure S1: The order that the highest ranked ape in each quartet took a stick, represented as percentage of trials.

### Model 5 (dyad)

Exploration of model 4 fit to dyad data.

Formula:

*Collapse Latency ~* 1 + *Proportion* * *Group Dominance Difference* * *Group Social Tolerance* + *Proportion*² + (1 | *Group ID*) + (1 | *Player name*)


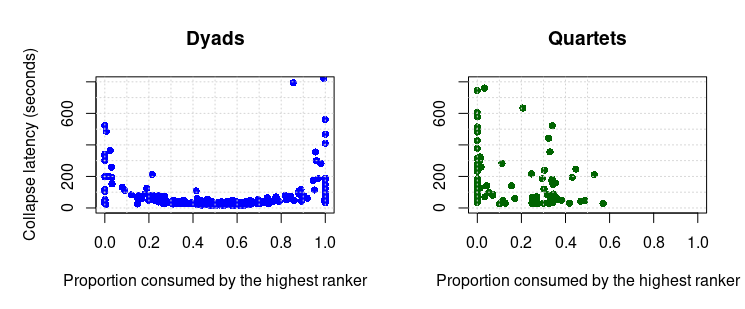


Figure S2: Visualisation of the U-shaped effect of *Proportion* in dyads, that was controlled for by the quadratic in Model 5.

| **Parameter** | **Estimate** | **Est. Error** | **95% CI (l, u)** | **Probability of direction (%)** | **Rhat** | **ESS** |
| --- | --- | --- | --- | --- | --- | --- |
| Intercept | 3.32 | 0.07 | 3.17, 3.45 | 100 | 1.00 | 2928 |
| Shape intercept | 1.52 | 0.08 | 1.35, 1.68 | 100 | 1.00 | 2914 |
| *Proportion* | 0.04 | 0.04 | -0.05, 0.12 | 78.95 | 1.00 | 3252 |
| *Group Dominance Difference* | -0.03 | 0.07 | -0.17, 0.11 | 68.75 | 1.00 | 2799 |
| *Group Social Tolerance* | -0.06 | 0.06 | -0.17, 0.06 | 85.02 | 1.00 | 3168 |
| *Proportion*² | 0.55 | 0.03 | 0.50, 0.61 | 100 | 1.00 | 2951 |
| *Proportion* : *Group Dominance Difference* | -0.08 | 0.06 | -0.18, 0.04 | 90.42 | 1.00 | 3095 |
| *Proportion* : *Group Social Tolerance* | 0.02 | 0.06 | -0.10, 0.14 | 64.30 | 1.00 | 3040 |
| *Group Dominance Difference: Group Social Tolerance* | 0.05 | 0.09 | -0.13, 0.24 | 71.20 | 1.00 | 2693 |
| Proportion : Group *Dominance Difference: Group Social Tolerance* | 0.03 | 0.09 | -0.15, 0.21 | 64.22 | 1.00 | 2954 |

| \|  \| \| --- \| |
| --- | --- |

Table S11:Model 5 summary table

**
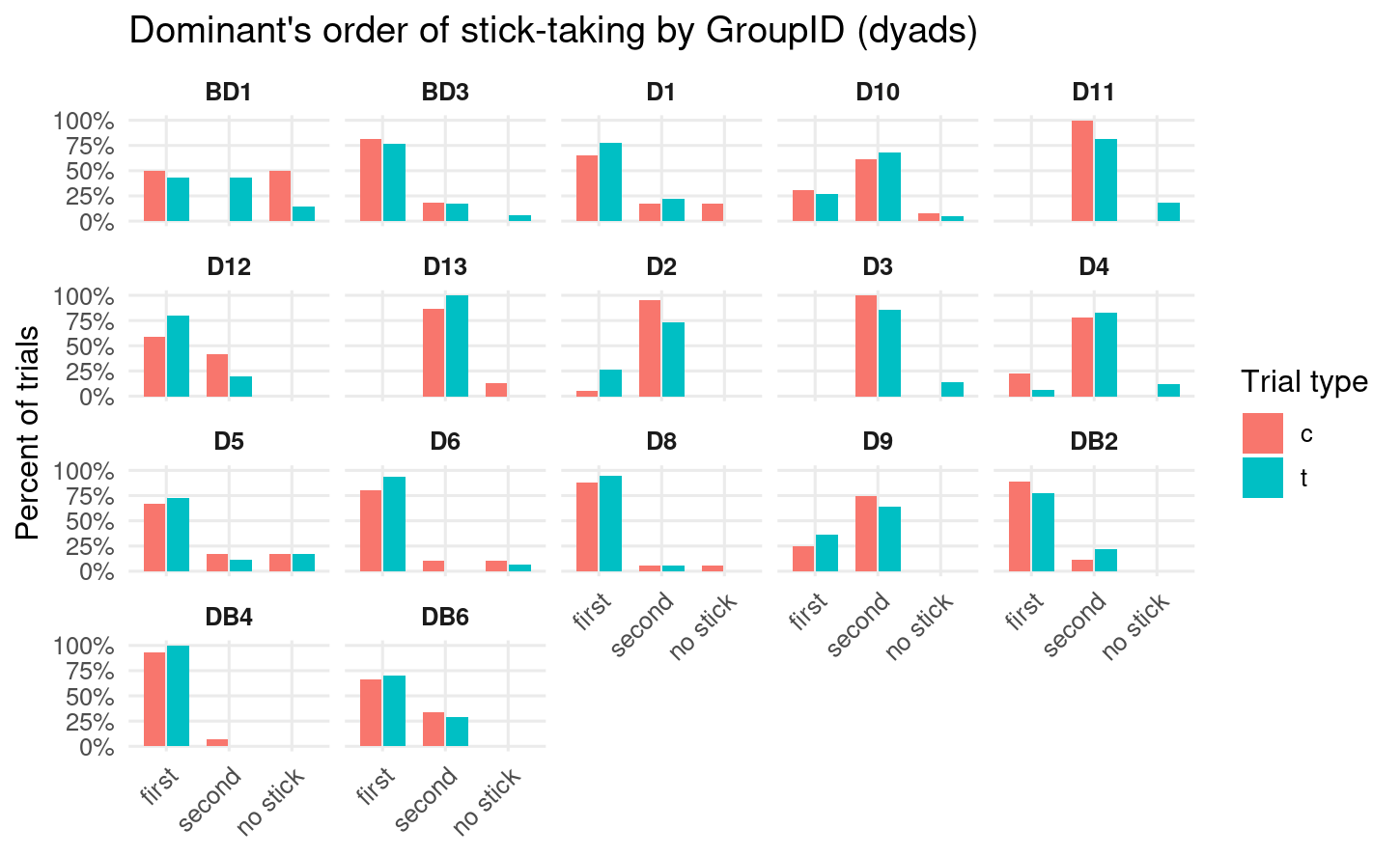
**

Figure S3: The order that the highest ranked ape in each dyad took a stick, represented as percentage of trials.

### Model 6

Addressing hypothesis 4:

Payoffs would be more unequal in dyadic groups than in quartets, and that this effect would be enhanced in groups that had asymmetrical dominance.

Formula:

*Normalised Entropy* ~ log(*Collapse* Latency) + *Group Size* + (1| *Group ID)* + (1 | *Player name*)

| **Parameter** | **Estimate** | **Est. Error** | **95% CI (l, u)** | **Probability of direction (%)** | **Rhat** | **ESS** |
| --- | --- | --- | --- | --- | --- | --- |
| Intercept | 0.00 | 0.16 | -0.31, 0.30 | 100 | 1.00 | 2052 |
| *log*(*Collapse Latency)* | -0.71 | 0.08 | -0.88, -0.54 | 100 | 1.00 | 2997 |
| *Group Size* | 1.03 | 0.29 | 0.45, 1.59 | 100 | 1.00 | 2217 |
| *Collapse Latency* : *Group Size* | 1.20 | 0.16 | 0.88, 1.52 | 100 | 1.00 | 3116 |
| *Phi* | 1.46 | 0.11 | 1.26, 1.69 | 100 | 1.00 | 3179 |
| ZOI | 0.12 | 0.02 | 0.09, 0.15 | 100 | 1.00 | 2917 |
| COI | 0.98 | 0.02 | 0.92, 1.00 | 100 | 1.00 | 1830 |

Table S12: Model 6 summary table

### Model 7 (dyads)

Addressing hypothesis 4:

Payoffs would be more unequal in dyadic groups than in quartets, and that this effect would be enhanced in groups that had asymmetrical dominance.

Formula:

*Normalised Entropy* ~ 1 + *Group Dominance Difference* + *Group Social Tolerance* + (1| *Group ID)* + (1 | *Player name*)

| **Parameter** | **Estimate** | **Est. Error** | **95% CI (l, u)** | **Probability of direction (%)** | **Rhat** | **ESS** |
| --- | --- | --- | --- | --- | --- | --- |
| Intercept | 3.32 | 0.07 | 3.17, 3.45 | 100 | 1.00 | 2928 |
| *Group Dominance Difference* | 0.10 | 0.17 | -0.24, 0.45 | 72.00 | 1.00 | 2588 |
| *Group Social Tolerance* | 0.12 | 0.16 | -0.18, 0.43 | 78.72 | 1.00 | 2546 |
| *Group Dominance Difference: Group Social Tolerance* | 0.02 | 0.22 | -0.41, 0.47 | 54.00 | 1.00 | 2597 |
| *Phi* | 0.66 | 0.05 | 0.56, 0.77 | 100 | 1.00 | 2759 |
| ZOI | 0.17 | 0.02 | 0.12, 0.21 | 100 | 1.00 | 2755 |
| COI | 0.98 | 0.02 | 0.92, 1.00 | 100 | 1.00 | 1872 |

Table S13: Model 7 summary table

### Model 8 (quartets)

Addressing hypothesis 5:

Payoffs would be more unequal in dyadic groups than in quartets, and that this effect would be enhanced in groups that had asymmetrical dominance.

Formula:

*Normalised Entropy* ~ 1 + *Group Dominance Difference* + *Group Social Tolerance* + (1| *Group ID)* + (1 | *Player name*)

| **Parameter** | **Estimate** | **Est. Error** | **95% CI (l, u)** | **Probability of direction (%)** | **Rhat** | **ESS** |
| --- | --- | --- | --- | --- | --- | --- |
| Intercept | 0.89 | 0.14 | 0.58, 1.16 | 100 | 1.00 | 2028 |
| *Group Dominance Difference* | -0.17 | 0.20 | -0.57, 0.24 | 80.90 | 1.00 | 2194 |
| *Group Social Tolerance* | -0.44 | 0.28 | -0.95, 0.17 | 93.40 | 1.00 | 2764 |
| *Group Dominance Difference: Group Social Tolerance* | 0.36 | 0.35 | -0.38, 1.00 | 85.97 | 1.00 | 2401 |
| *Phi* | 12.31 | 1.68 | 9.27, 15.82 | 100 | 1.00 | 2989 |
| ZOI | 0.01 | 0.01 | 0.00, 0.03 | 100 | 1.00 | 2096 |
| COI | 0.50 | 0.29 | 0.03, 0.97 | 100 | 1.00 | 2467 |

Table S14: Model 8 summary table

## Other statistical methods

As a preliminary assessment of the measure to be used to calculate *proportion*  in model 4, a Spearman’s rank correlation was run to assess corrlation between the of scoops eaten by the individuals in a trial and the duration of time they held a stick. The two measures were found to be highly correlated (*r_s_*=0.956, *p*=<0.001, *n*=103).

We also tested the correlation between *Last Stick Latency* and *Collapse Latency* in test condition trials (*N*=374). Very high correlation was found (*r_s_*=0.98, *p*=<0.001, *n*=374) (see fig S4).

**
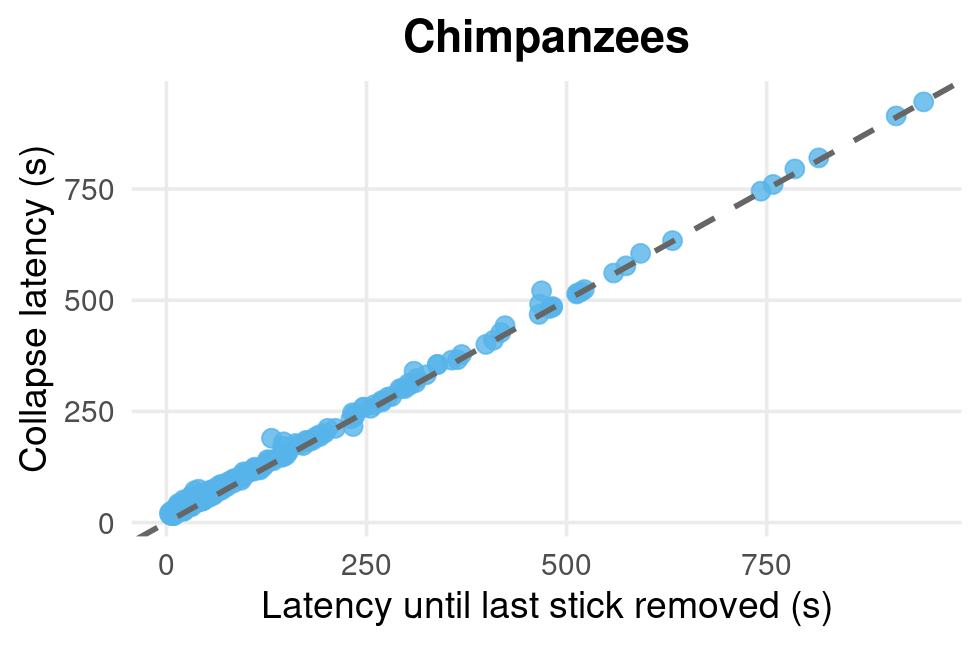
**

Figure S4: *Collapse Latency* and *Last Stick Latency* in test condition trials were strongly correlated.

Kendall’s coefficient of concordance was used to test agreement between dominance rank ratings, finding a strong agreement between the four raters of the 10 A-group chimpanzees (W=0.86, χ²(8)=27.6, p=0.001) and between the three raters of the 5 B-group chimpanzees (W=0.96, χ²(4)=11.5, p=0.022).

# Intervention behaviours

Discrete behaviours that may indicate strategic play include guarding of sticks, grabbing sticks from the hands of other players, and touching or obstructing players removing sticks. We also recorded instances where a participant vocalised in response to or in anticipation of sticks being moved. These “intervention behaviours” are not reported in the main text due to their low rates of occurrence, however, the observed higher rates of these behaviours in the test compared to the control further support that the participants perceive the social dilemma and non-social dilemma conditions differently and actively engage in behaviours that promote resource sustainability.

### Stick guarding

Stick guarding was defined in the ethogram as “Focal places their hand on stick 1 still inside mesh (NOT moving it around to try and gather yoghurt. Simply holding it in place)”. This behaviour was an effective way of preventing resource collapse as it deterred other apes from removing the guarded stick, which would contribute to over extraction of the resource. This behaviour was never observed in the control condition (table S15).

| **Duration of guarding (s)** | **Group size** | **Group ID** | **Condition** | **Trial type number** |
| --- | --- | --- | --- | --- |
| 8.24 | 4 | Q3 | test | 2 |
| 19.74 | 4 | Q3 | test | 8 |
| 120.76 | 4 | Q3 | test | 16 |
| 48.56 | 4 | BQ2 | test | 3 |
| 575.00 | 4 | BQ2 | test | 8 |
| 2.16 | 4 | Q1 | test | 15 |
| 0.96 | 2 | BD2 | test | 11 |

Table S15: Details of stick guarding observations. In 704 trials, this behaviour was observed in 7 trials, always in the test condition.

### Physical interventions

Physical intervention was defined as “Focal touches or obstructs another player taking a stick”. In 704 trials, it was observed once in the control condition and three times in the test (table S16).

|  | **dyad** | **quartet** |
| --- | --- | --- |
| Control | 1 | 0 |
| Test | 1 | 2 |

Table S16: Details of physical interventions. Count occurance out of 704 trials in total.

### 4.3 Vocal interventions

Intervention vocalisation was defined as “focal vocalises in response to another player taking a stick”. It was observed twice in the control condition and seven times in the test (table S17).

|  | **dyad** | **quartet** |
| --- | --- | --- |
| Control | 0 | 2 |
| Test | 4 | 3 |

Table S17: Details of intervention vocalisations. Count occurance out of 704 trials in total.

### Stick grabbing

Stick grabbing was defined as “Focal appropriates the stick from another player or attempts to grab it from them”. This was an effective way for a participant to access the yoghurt without removing a stick and pushing the resource closed towards collapse. It was observed 6 times in the control condition and 11 times in the test condition (table S18).

|  | **dyad** | **quartet** |
| --- | --- | --- |
| Control | 2 | 4 |
| Test | 2 | 9 |

Table S18: Details of stick grabbing. Count occurance out of 704 trials in total.

# Supplementary References

Bürkner, P.-C. (2017). brms: An R Package for Bayesian Multilevel Models Using Stan. *Journal of Statistical Software*, *80*, 1–28. https://doi.org/10.18637/jss.v080.i01

Koomen, R., & Herrmann, E. (2018). Chimpanzees overcome the tragedy of the commons with dominance. *Scientific Reports*, *8*(1), Article 1. https://doi.org/10.1038/s41598-018-28416-8

Makowski, D., Luedecke, D., Patil, I., Thériault, R., Den-Shachar, M. S., & Wiernik, B. (2023). Automated Results Reporting as a Practical Tool to Improve Reproducibility and Methodological Best Practices Adoption. *CRAN*. https://easystats.github.io/report/

Schweinfurth, M. K., Detroy, S. E., Van Leeuwen, E. J. C., Call, J., & Haun, D. B. M. (2018). *Spontaneous social tool use in chimpanzees (Pan troglodytes)*. https://doi.org/10.1037/com0000127.supp

Vehtari, A., Gelman, A., Simpson, D., Carpenter, B., & Bürkner, P.-C. (2021). Rank-Normalization, Folding, and Localization: An Improved Rˆ for Assessing Convergence of MCMC (with Discussion). *Bayesian Analysis*, *16*(2). https://doi.org/10.1214/20-BA1221

Völter, C. J., Rossano, F., & Call, J. (2015). From exploitation to cooperation: Social tool use in orang-utan mother–offspring dyads. *Animal Behaviour*, *100*, 126–134. https://doi.org/10.1016/j.anbehav.2014.11.025
